# Revealing the impact of 17 mutations of human FMO3 protein associated with trimethylaminuria on its local spatial properties: a bioinformatic approach

**DOI:** 10.1186/1753-6561-9-S1-A59

**Published:** 2015-01-14

**Authors:** TV Borodina, SE Vakal

**Affiliations:** 1 ESC, Institute of Biology, Taras Shevchenko National University of Kyiv, Ukraine

## Background

Trimethylaminuria (TMAU) is a rare metabolic disorder manifesting in enormous excretion of trimethylamine (TMA) with urea, sweat and breath that leads to unpleasant body odour similar to rotting fish. TMAU has a strong genetic basis: 18 mutations (associated with 17 amino acid substitutions or chain truncation) of flavin-containing monooxygenase 3 (FMO3) are now recognized as a causative factor of TMAU. Surprisingly, only few of them are related with active site structure, while the molecular basis of other mutations impact on the protein structure is unknown. Moreover, there are no FMO3 models solved experimentally. So, the aim of study was to reveal the effects of 17 known mutations on human FMO3 structure by means of structural bioinformatics techniques.

## Methods

Full-size modelling of normal FMO3 structure was performed with multiple template-based homology modelling and fragment threading techniques in MODELLER and FUGUE software. Point mutations were created with special MODELLER script and DeepView in parallel. Geometry optimization was performed with GROMOS96 force field. Binding sites were identified with Q-Site Finder and SURFNET. List of mutations were taken from Zhou and Shephard paper. Structural analysis of FMO3 normal and TMAU-associated structures was performed with UCSF Chimera. Aggregation tendency was calculated with PASTA and TANGO. All structures visualization was performed with PyMOL.

## Results

Full-size structures of normal and 17 TMAU-associated FMO3 were obtained for the first time by homology modelling based on 4 templates and C-terminal domain threading. Significant changes in electrostatic potential and force-field energy distribution were fixed for few point mutations (e.g., R51G, E158K etc.). Disturbance of few conservative and functionally important residues environment were established for R51G, N61S, M66I, E308G, M434I etc. Nine point-mutations were found to destabilize binding pocket of FMO3 thus inhibiting its possibility to catalyze TMA oxidation. However, exposed residues are mutated in more cases, than buried. No clear impact on NADP binding efficacy and aggregation probability of mutated variants in comparison with normal FMO3 were determined. Analysis of three mutations (E24D, E158K, V257M) associated with high FMO3 activity showed that slight redistribution of local electrostatic potential near few conservative residues can increase catalytic activity, thus suggesting the direction for future drugs investigation.

## Conclusions

1) Full-size structures of human normal and TMAU-associated FMO3 protein are obtained; 2) Most of TMAU-associated mutations possess local destabilizing effect on spatial structure; 3) NADP binding and aggregation tendency aren’t usually affected; 4) Surface-stabilizing ligands for FMO3 are potential drugs for TMAU treatment (next steps).

